# Mutant GDF5 enhances ameloblast differentiation via accelerated BMP2-induced Smad1/5/8 phosphorylation

**DOI:** 10.1038/srep23670

**Published:** 2016-03-31

**Authors:** Jia Liu, Kan Saito, Yuriko Maruya, Takashi Nakamura, Aya Yamada, Emiko Fukumoto, Momoko Ishikawa, Tsutomu Iwamoto, Kanako Miyazaki, Keigo Yoshizaki, Lihong Ge, Satoshi Fukumoto

**Affiliations:** 1 Department of Pediatric Dentistry, Peking University School and Hospital of Stomatology, Beijing 100081, China; 2 Division of Pediatric Dentistry, Department of Oral Health and Development Sciences, Tohoku University Graduate School of Dentistry, Sendai 980-8575, Japan; 3 Department of Pediatric Dentistry, The First Affiliated Hospital of Xinjiang Medical University, Urumqi 830054, Xinjiang, China; 4 Division of Pediatric Dentistry and Special Needs Dentistry, Department of Developmental Oral Health Science, Iwate Medical University, Morioka 020-8505, Japan; 5 Section of Orthodontics and Dentofacial Orthopedics, Department of Oral Health, Growth and Development, Kyushu University Faculty of Dental Science, Fukuoka 812-8582, Japan

## Abstract

Bone morphogenetic proteins (BMPs) regulate hard tissue formation, including bone and tooth. Growth differentiation factor 5 (GDF5), a known BMP, is expressed in cartilage and regulates chondrogenesis, and mutations have been shown to cause osteoarthritis. Notably, GDF5 is also expressed in periodontal ligament tissue; however, its role during tooth development is unclear. Here, we used cell culture and *in vivo* analyses to determine the role of GDF5 during tooth development. GDF5 and its associated BMP receptors are expressed at the protein and mRNA levels during postnatal tooth development, particularly at a stage associated with enamel formation. Furthermore, whereas BMP2 was observed to induce evidently the differentiation of enamel-forming ameloblasts, excess GDF5 induce mildly this differentiation. A mouse model harbouring a mutation in GDF5 (W408R) showed enhanced enamel formation in both the incisors and molars, but not in the tooth roots. Overexpression of the W408R GDF5 mutant protein was shown to induce BMP2-mediated mRNA expression of enamel matrix proteins and downstream phosphorylation of Smad1/5/8. These results suggest that mutant GDF5 enhances ameloblast differentiation via accelerated BMP2-signalling.

The development of many ectodermal organs, such as teeth, hair, and exocrine glands, is initiated by reciprocal interactions between the mesenchyme and epithelium that modulate cellular proliferation, differentiation, and apoptosis in these tissues. Most organs originating from the ectoderm form via a similar thickening of the epithelium into a placode utilising similar mechanisms. Notably, the differentiation of an epithelial placode into an ectodermal organ appears to be largely controlled by stimulation from the adjacent mesenchyme through the secretion of various factors. In particular, teeth are hard tissues composed of layers of enamel and dentin around the interior dental pulp. After the odontoblasts form an initial layer of dentin, ameloblasts differentiate from the inner dental epithelium and begin secreting the enamel matrix proteins amelogenin. (AMEL), ameloblastin (AMBN), and enamelin. The process of amelogenesis involves a number of essential factors, including AMBN, a cell adhesion molecule essential for ameloblast binding^1^, and amelogenin, the major extracellular matrix molecule produced by ameloblasts, both of which can be used as markers of ameloblast differentiation. Furthermore, in addition to basic amelogenesis, the formation of molar cusps creates an additional layer of complication that involves numerous regulatory proteins. Various growth factors, e.g., transforming growth factors (TGFs) and extracellular matrix proteins, have been shown to play important roles in determining tooth size and shape during these processes^2^,^3^,^4^,^5^,^6^.

The TGF superfamily is divided into multiple subfamilies, which include activins, growth differentiation factors (GDFs), and bone morphogenetic proteins (BMPs)^7^. In teeth, activin A has been shown to be an essential component of incisor and mandibular molar development, as these teeth fail to develop past the bud stage in activin A knockout (KO) mice^8^. Furthermore, the activin-binding protein follistatin appears to play a role in enamel formation and shaping of the tooth crown^9^. BMPs, originally identified for their heterotopic bone-inducing activity in bone matrix^10^,^11^, also appear to play key roles during tooth development^12^. For example, odontoblast differentiation is regulated by BMP2, which has been detected in the dental epithelium, dental papilla, and secretory odontoblasts^13^,^14^,^15^. BMP4 expression is also observed in the dental epithelium during the early stages of tooth development, where it mediates epithelial-mesenchymal interactions, but this expression later shifts to the mesenchyme during placode formation^16^. Pre-odontoblast and pre-ameloblast expression of BMP7 was also previously reported^17^. In the context of dental development, GDF5, 6, and 7 have been detected in the periodontal tissues, dental follicle, and odontoblasts^18^. GDF5 (also known as BMP14), which is also expressed in the mesenchyme of primordial cartilage during early limb development^19^,^20^,^21^, has been postulated to have a function related to proliferation, differentiation, and angiogenesis during bone and cartilage formation^20^,^22^,^23^, indicating a possible role in similar processes during tooth development.

BMPs are known to function by binding to their type I and type II membrane receptors (BMPRI and BMPRII, respectively). Type I receptors, such as BMPRIA (ALK3) and BMPRIB (ALK6), propagate signal transduction by phosphorylating Smad proteins 1, 5, and 8 (Smad1/5/8), which then form a trimeric complex with Smad4 and translocate into the nucleus^24^,^25^. Thus, the Smad1/5/8 and Smad4 pathways appear to be essential during BMP signalling. Furthermore, BMPRIA expression has been detected in epithelial cells, the stratum intermedium, and the dental papilla, whereas BMPRIB appears to be primarily expressed in the inner enamel epithelium and by odontoblasts. Expression of type II receptors has been observed in the stratum intermedium and the inner enamel epithelium^26^,^27^. The essential function of BMPRIA was previously highlighted by a study using a conditional KO model, where the incisor and molar teeth were absent in newborn mice, as they failed to develop past the bud stage^28^. BMPRIB has also been implicated in ameloblast differentiation in association with odontogenic ameloblast-associated protein (ODAM)^29^. Thus, although these receptors and their various growth factor ligands appear to play critical functions during tooth development, the present understanding of their full functions and interactions during this process is limited.

GDF5 has high binding affinity to BMPRIB, BMPRII^30^ and Activin type II receptors^31^. Recently, an alternative pathway has been suggested to occur during chondrogenesis in which both GDF5 and BMP2 bind to type I receptors, and the ternary complex changes the activity of type II receptor binding^32^. The role of GDF5-BMPRII binding is further complicated by the variation in calcification observed for known GDF5 point mutations, such as W414R^33^, N445T^34^, N445K^34^, and S475N^32^. GDF5 also act as neurotrophic factor and induced neuronal cell differentiation through a smad dependent pathway^35^,^36^. Furthermore, the neurotrophin NT-4 induced ameloblast differentiation^37^,^38^, indicating that neurotrophic effect of growth factors is important for ameloblast differentiation. However, the full effects of GDF5 on ameloblast differentiation are not currently clear.

In the present study, we sought to elucidate the function of GDF5 in the cellular context of tooth development using cell culture techniques in parallel with an *in vivo* mouse model harbouring a dominant negative W408R mutation in GDF5. To our knowledge, this is the first article in which GDF5 has been indicated to function not only in cartilage and bone mesenchyme, but also during epithelial-to-ameloblast differentiation.

## Results

### GDF5 and BMPRs are expressed in various areas of the tooth germ at specific developmental stages

**Figure 1:**
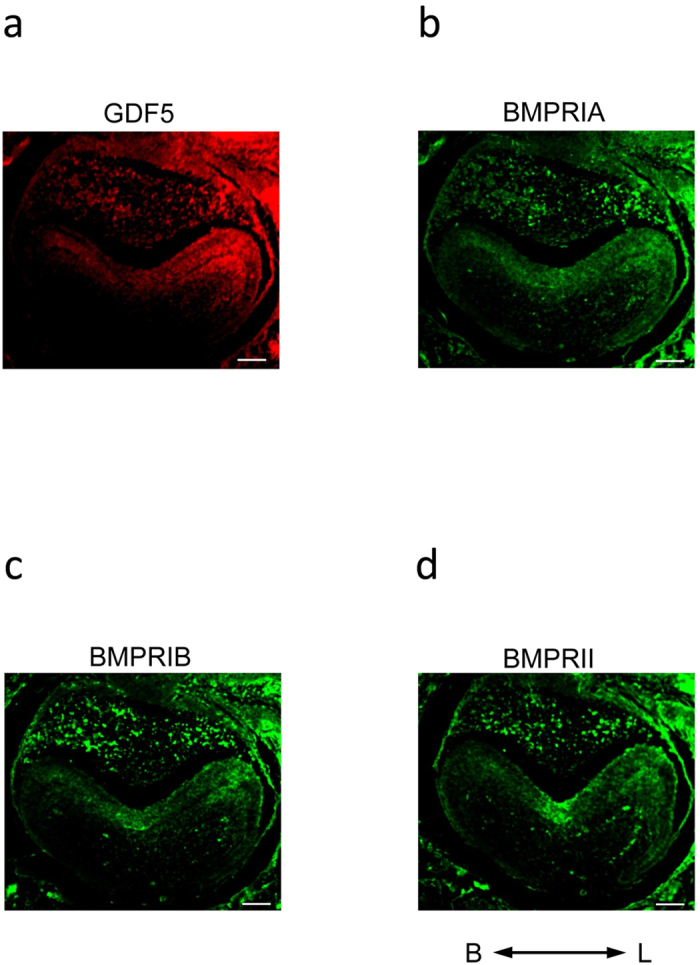
Expression pattern of GDF5 and various BMPRs in molar tooth germ cells. Sections of the molar of E18 mouse were immunostained for GDF5 (**a**), BMPRIA (**b**), BMPRIB (**c**), and BMPRII (**d**). Scale bars: 200  μm. Abbreviations: B, Buccal side; L, Lingual side.

BMPs are expressed mainly in the mesenchyme, and they regulate differentiation of the epithelium via the BMPRs present on the epithelial surface. However, the expression pattern of GDF5 in the dental epithelium is largely unknown. Thus, we first evaluated the expression of GDF5 in addition to BMPRIA, BMPRIB, and BMPRII using immunohistochemical staining of the murine molar tooth germ at 1 day postnatal (Fig. 1). Expression of GDF5 was detected in the stellate reticulum and ameloblast cells. GDF5 was strongly expressed in the ameloblasts present in the lingual cusp. BMPRIA and BMPRIB were also weakly expressed in the stellate reticulum and ameloblast cells. In contrast, BMPRII was observed in the stellate reticulum and the ameloblasts present in the grooved region, excluding the enamel knot.

**Figure 2:**
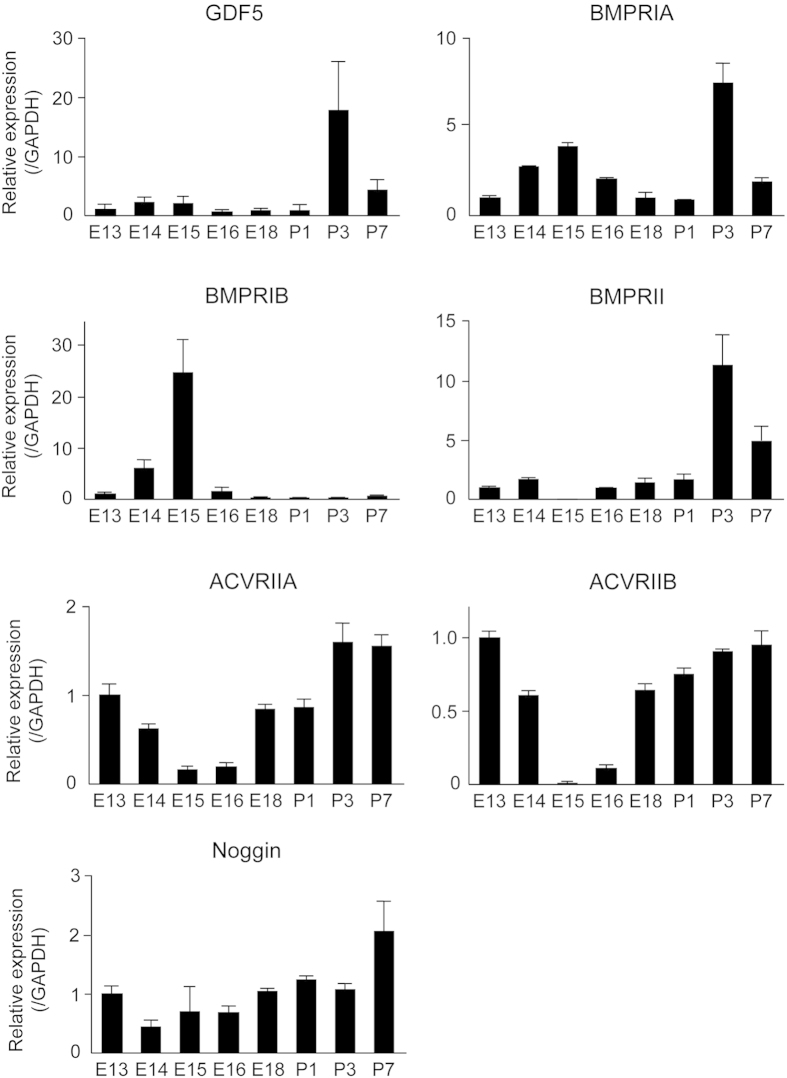
Expression of GDF5, BMPRIA, BMPRIB, BMPRII, ACVRIIA, ACVRIIB and noggin at various stages of tooth development. Expression of GDF5 and various BMPRs, ACVRs was measured in the molar tooth germ cells from embryonic day 13 (E13) to 7 days postnatal (P7) using real-time PCR. Expression of each gene was normalised to that of GAPDH, and the relative expression at each time point was determined based on the expression at E13 (set to 1.0).

In addition to investigating the protein expression pattern in postnatal molars, we also sought to determine the relative mRNA expression of these genes at various stages of development. Thus, samples of molar tooth germ were isolated from various stages, beginning at embryonic day 13 (E13) through 7 days postnatal (P7), and the expression of GDF5, BMPRs including Activin receptors (ACVRs) and Noggin was analysed by real-time PCR (Fig. 2 and Supplemental figure a). The expression of BMPRIA, BMPRII, and GDF5 appeared to increase mainly during postnatal tooth development, with the peak expression being observed at P3. In contrast, BMPRIB was strongly expressed at E15. Activin receptor IIA and B (ACVRIIA and ACVRIIB) were expressed in the early embryonic stage and postnatal days, except E15 and 16. Noggin expression increased in later stages of tooth development. These results indicate that the expression of each BMPR differs, probably in both localisation and peak stage, depending on the developmental stage of the tooth. Our data also highlight the similarity in BMPRIB and GDF5 localisation, whereas the temporal expression pattern of GDF5 was more similar to that of BMPRII. In contrast, both the localisation and expression pattern were aligned for GDF5 and BMPRIA. Thus, we consider that GDF5 does in fact influence tooth differentiation via these BMPRs.

### Ameloblast differentiation was activated following stimulation with BMP2 in SF2 cells

**Figure 3:**
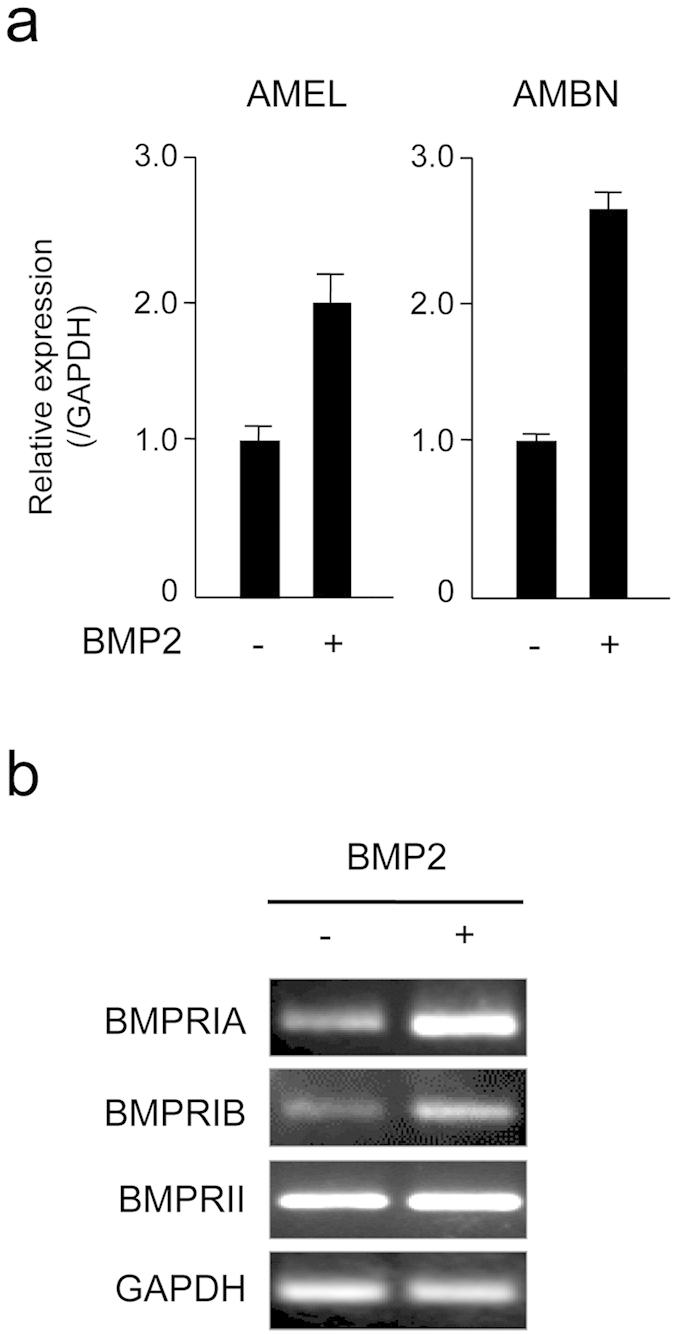
Influence of BMP2 on the expression of amelogenin (AMEL), ameloblastin (AMBN), and various BMPRs in SF2 cells. SF2 cells were cultured with BMP2, and the expression of AMEL and AMBN was analysed by real-time PCR (**a**), whereas the expression of BMPRIA, BMPRIB, and BMPRII was determined by RT-PCR (**b**). The expression of all genes analysed was normalised to the expression of GAPDH as an internal control.

Although GDF5 and the various BMPRs were found to be expressed *in vivo*, for a full analysis of the interaction and function of these proteins, it was also necessary to utilise cell culture techniques. Although the rat dental epithelium SF2 cell line was previously established in our laboratory^38^, it is not clear whether ameloblast differentiation is activated by BMP in these cells or if they even express the same BMPRs present in the primary dental epithelium. Thus, we sought to analyse the mRNA expression of AMEL and AMBN, two ameloblast differentiation markers, in BMP2-stimulated SF2 cells using RT-PCR. Addition of BMP2 to the culture medium induced expression of both AMEL and AMBN (Fig. 3a and Supplemental figure b). The RT-PCR method was utilised to evaluate the expression of the three BMPRs in the SF2 cells. BMPRIA and BMPRIB were only expressed at low levels, whereas BMPRII was strongly expressed in this epithelial cell type (Fig. 3b). Notably, following BMP2 stimulation, expression of BMPRIA and BMPRIB mRNA increased. These BMPRs therefore facilitate the BMP2-mediated increase in ameloblast differentiation in this dental epithelial cell line. These results also suggest that SF2 cells can be utilised to analyse BMP signalling during tooth development.

### Excess GDF5 inhibits BMP2-induced amelogenin and ameloblastin expression

**Figure 4:**
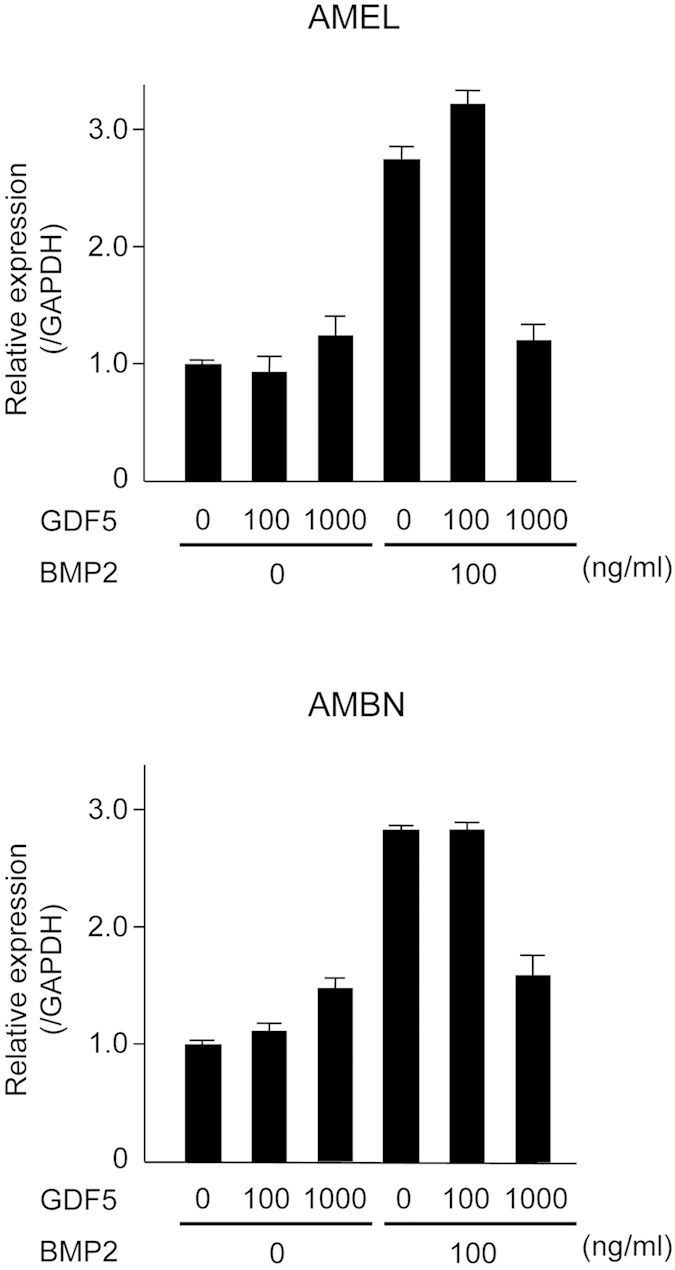
Influence of BMP2 and GDF5 on ameloblast differentiation. SF2 cells were incubated with various combinations of BMP2 (100 ng/ml) and GDF5 (100 or 1000 ng/ml), and the expression of amelogenin (AMEL) and ameloblastin (AMBN) was analysed with real-time PCR.

As GDF5 and BMPRs were shown to be expressed in ameloblasts, it is plausible that GDF5 may regulate ameloblast differentiation in a manner similar to that of BMP2. Therefore, stimulation of ameloblast differentiation following the addition of BMP2 and/or GDF5 was assessed. Similar to our earlier results, BMP2 induced expression of both AMEL and AMBN (Fig. 4 and Supplemental figure b). However, when stimulation was performed with excess GDF5 without BMP2, major changes were not observed in AMBN expression, whereas AMEL showed slight induction. In particular, 100 ng/ml GDF5 did not affect AMEL and AMBN expression induced by BMP2. However, 1000 ng/ml GDF5 inhibited BMP2-induced AMEL and AMBN expression. These results suggest that the effect of GDF5 on BMP2-induced ameloblast differentiation was concentration-dependent.

### W408R mutation in GDF5 causes hypercalcinosis in the enamel of affected mice

**Figure 5:**
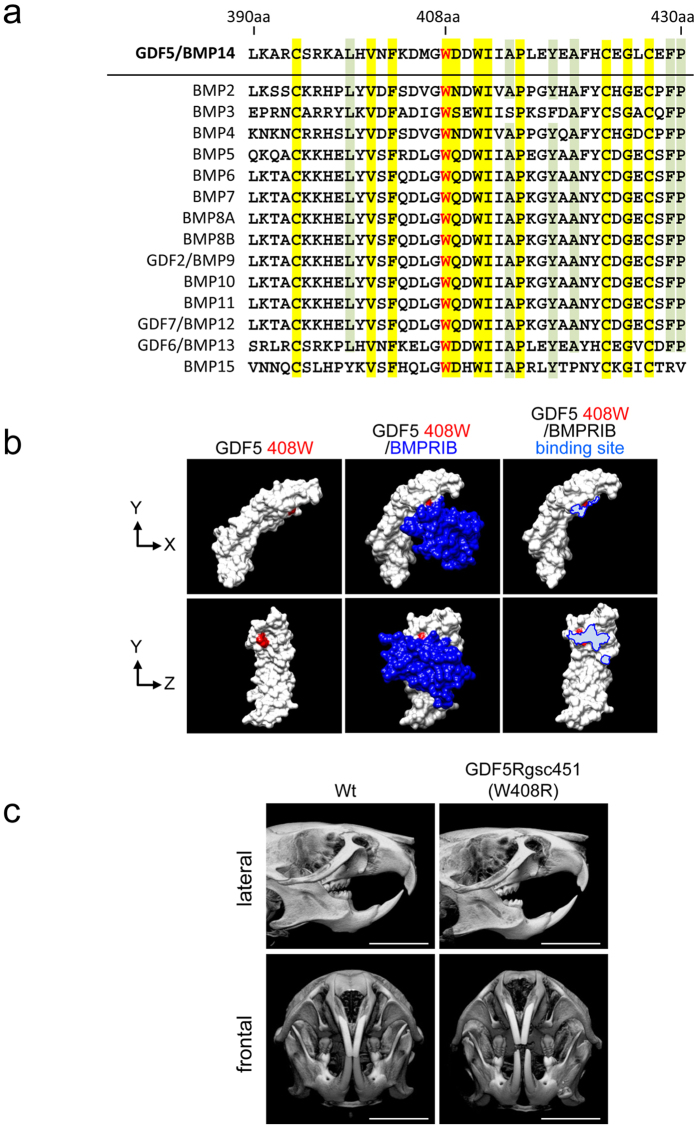
Structural analysis of GDF5 and micro-CT imaging of W408R GDF5 mutant and wild-type mice. (**a**) Multiple protein sequence alignment of BMP family. Integrity identity; yellow. High similarity; green. (**b**) The tertiary structure was assembled using the mouse GDF5 sequence alignment. Amino acid 408 is highlighted in red. (**c**) Wild-type and GDF5^Rgsc451^ (W408R) mice were evaluated using a micro-CT scanner. Scare bar: 5,000 μm.

**Figure 6:**
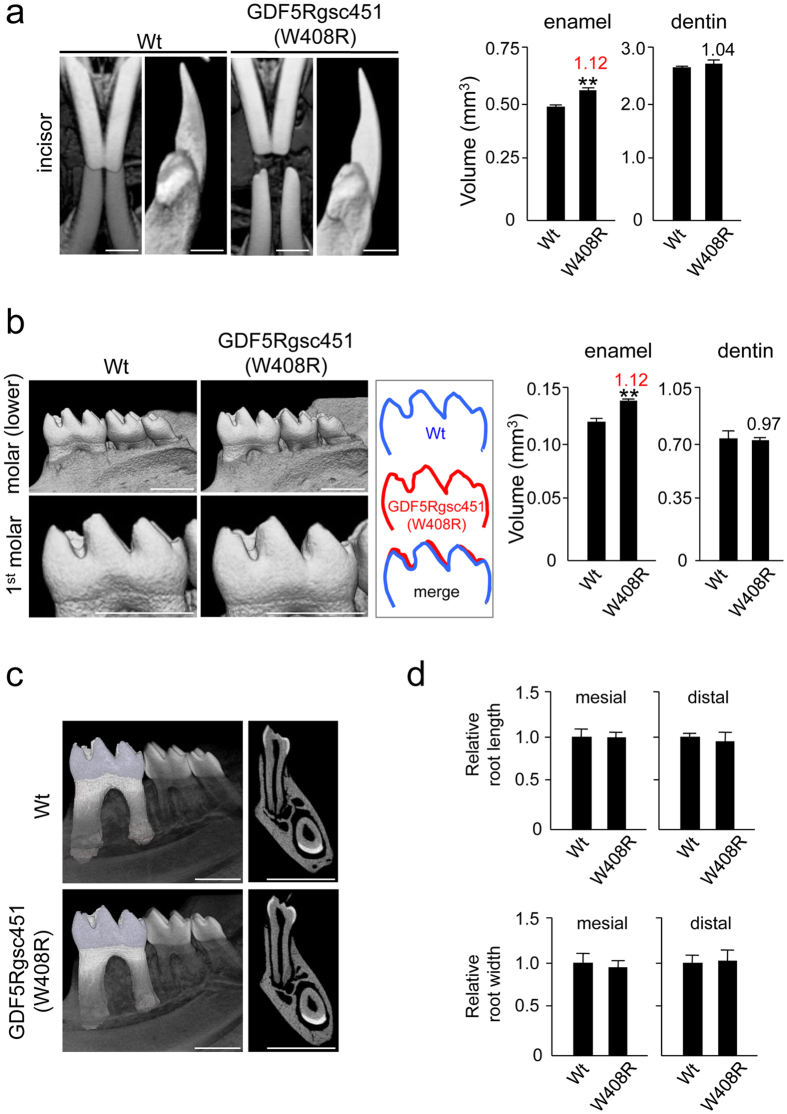
Micro-CT analysis of W408R mutant and wild-type mice and determination of various tooth parameters. (**a**) High-magnification CT image of wild-type and mutant incisors (left). The volumes of the enamel and dentin were then calculated (right). The value provided above the W408R bar is the volume relative to the wild type. (**b**) High-magnification images of wild-type and mutant molars (left). The schemes of the enamel were traced (highlighted in blue and red). The molar enamel capacity and the relative volume (compared to the wild type) value were then calculated (right). (**c**) Sectional micro-CT images of wild-type and mutant mice. (**d**) Length and width of the mesial and distal roots in wild-type and mutant molars. Scare bars: 1,000 μm. **P < 0.05.

The murine GDF5 protein consists of 495 amino acids, of which the N-terminus accounts contains 27 as a signal sequence, whereas the C-terminus contains 120 amino acids to form the mature peptide. The homology between the mature GDF5 protein and BMP2 is 52% (Data not shown). Moreover, amino acids 407 and 408 (W-G region) are highly conserved across the BMP family (Fig. 5a). According to our analysis of the tertiary structure, amino acid 408 of mouse GDF5 was located on the protein surface (Fig. 5b). Analysis of the binding site of GDF5 and BMPRIB (Fig. 5b) revealed that it is important for binding to BMPRIB because 408 W was present in the binding region. To evaluate the function of this particular amino acid residue, we evaluated GDF5 function using mice harbouring the W408R point-mutation. As previously reported^39^, slight hypomineralisation was observed in several cranial bones (Fig. 5c), including the sphenoid bone, zygomatic arch, and mandibular condyle. In contrast, the mandibular incisor of the W408R mouse was clearly white in comparison to the wild type (Fig. 6a), suggesting that the enamel calcification of the incisor was accelerated, while that of the dentin was unaffected. Enamel formation was also accelerated in the molars of the W408R mouse, which showed signs of hypertrophy (Fig. 6b). Notably, these effects were not observed in the calcification of the alveolar bone or root, which has been associated with various periodontal diseases (Fig. 6c). The effects of the W408R point mutation were also limited in terms of changes in the length and width of the root (Fig. 6d). Furthermore, in histological observation, there was no phenotypic alteration including morphology of tooth germ and cell polarity in both ameloblasts and odontoblasts in GDF5 W408R mice compared with wild-type mice (data not shown). These data indicate that although bone calcification decreased in the mesenchyme, there was no substantial effect of the W408R mutation on root formation. In contrast, hyperplasia was observed in the epithelial-derived enamel. Thus, it is suggested that the effects of the W408R GDF5 are different in the mesenchyme and epithelium.

### The W408R GDF5 mutation strongly induces ameloblast differentiation

**Figure 7:**
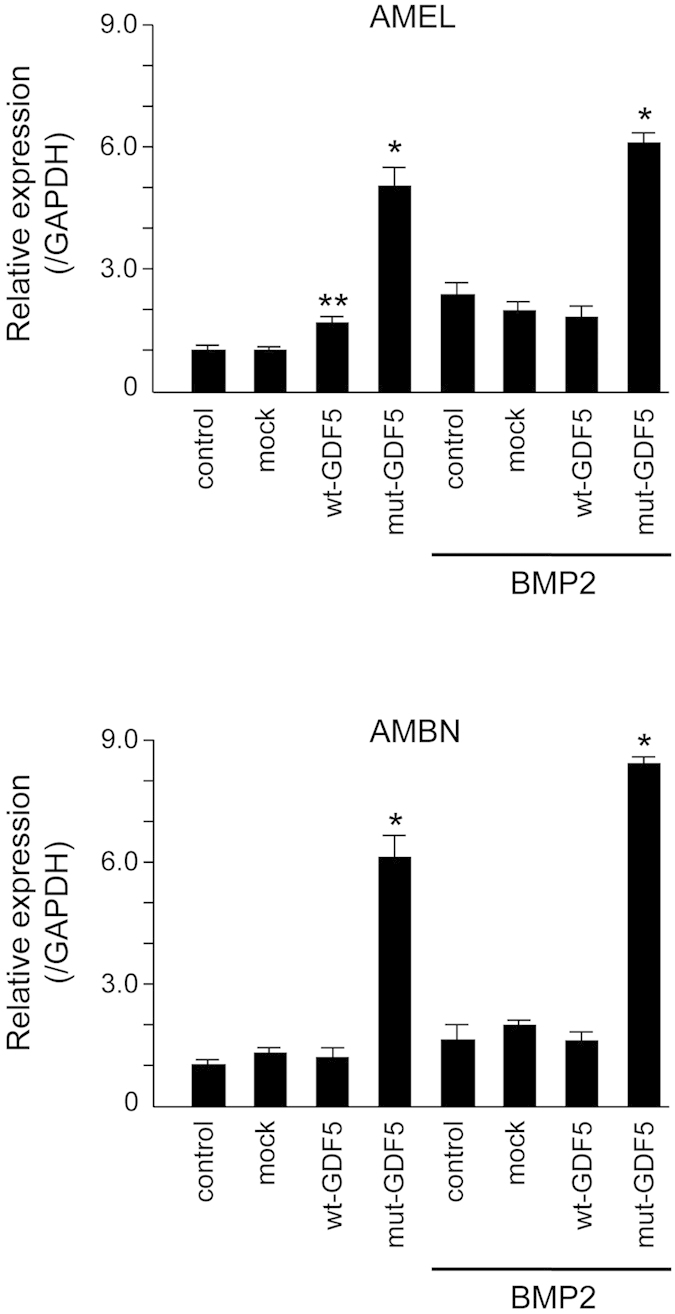
Influence of various combinations of BMP2 and wild-type or mutant GDF5 on ameloblast differentiation. SF2 cells transfected with no plasmid (control), mock expression plasmid, wild-type GDF5, or mutant GDF5 were cultured with BMP2, and the expression of amelogenin (AMEL) and ameloblastin (AMBN) was evaluated by real-time PCR. Expression was normalised to that of GAPDH, and the control sample without BMP2 was considered the baseline and set at 1.0, from which the relative expression for every other condition was compared. *P < 0.01, **P < 0.05.

To further evaluate the mechanisms underlying the novel changes observed in enamel calcification in the W408R GDF5 mutant mouse, we utilised SF2 cell culture transfection using wild type (wt-GDF5) or W408R mutant GDF5 (mut-GDF5). The transfected cells were cultured with and without BMP2, and the expression of AMEL and AMBN was used to evaluate differentiation (Fig. 7). Similar to our earlier results, the expression of AMEL was weakly increased by wild-type GDF5 without BMP2, but ameloblast differentiation was not induced by wt-GDF5 with BMP2. However, following transfection with the W408R GDF5 mutant plasmid, expression of both AMEL and AMBN increased, and this increase was further enhanced by addition of BMP2. These data suggest that W408R GDF5 may activate the BMP signalling pathway in epithelial cells, but not in the mesenchyme.

### W408R GDF5 activates Smad1/5/8 phosphorylation in epithelial cells

**Figure 8:**
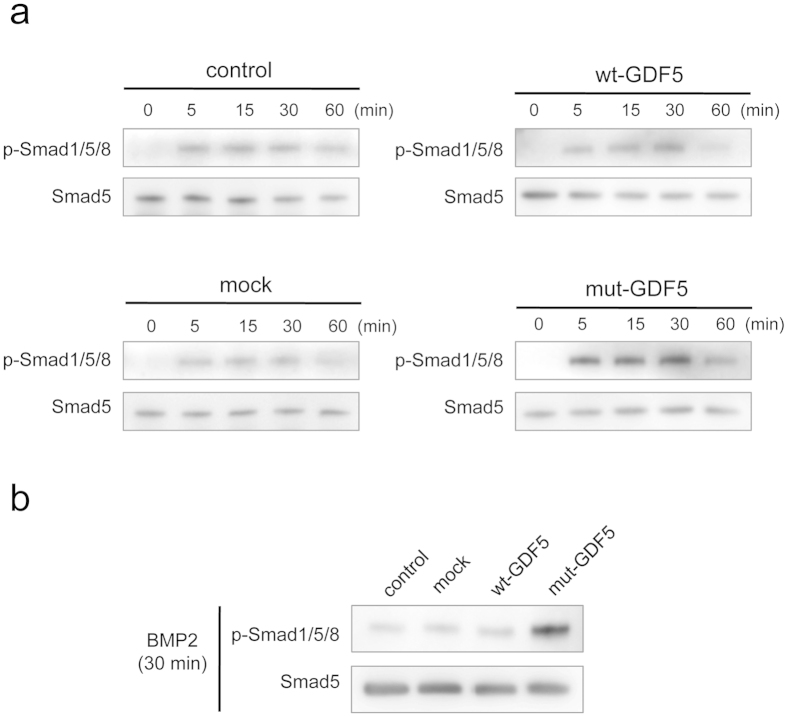
Effect of various combinations of BMP2 and wild-type or mutant GDF5 on Smad1/5/8 signalling. SF2 cells transfected with no plasmid (control), mock expression plasmid, wild-type GDF5, or mutant GDF5 for 0, 5, 15, 30, or 60 min were analysed by Western blot using anti-Smad5 or anti-phospho Smad1/5/8 antibodies (**a**). Similarly, cells transfected with no plasmid (control), mock expression plasmid, wild type GDF5, or mutant GDF5 were also stimulated for 30 min with BMP2, and the expression of Smad5 and phospho-Smad1/5/8 was evaluated (**b**).

Whereas TGF is known to induce phosphorylation of Smad2/3, BMPs and GDFs have been shown to induce intracellular phosphorylation of Smad1/5/8. Therefore, we next evaluated the downstream phosphorylation of Smad1/5/8 in SF2 cells transfected with mock vector or wild-type and W408R mutant GDF5 following BMP2 stimulation for the designated time-period (Fig. 8a). Notably, Smad1/5/8 phosphorylation was not detected in transfected cells before BMP activation (time 0). However, Smad1/5/8 was phosphorylated in all transfected cells after incubation with BMP2 for 5 to 30 min, after which the phosphorylation levels decreased. To directly compare the level of Smad 1/5/8 phosphorylation in all transfected cells at 30 min of BMP2 incubation, we analysed samples on the same blot (Fig. 8b). These results suggest that although W408R GDF5 alone may not influence phosphorylation of Smad1/5/8, it does appear to augment stimulation of BMP2 in epithelial cells. It was considered that this increase in phosphorylation, and thus downstream activity, in the dental epithelium is likely the cause of the enhanced enamel calcification phenotype observed in the W408R GDF5 mutant mouse.

## Discussion

BMPs, in addition to their function in bone morphogenesis, also play roles in brain, eye, hair follicle, kidney, lung, liver, skin, and tooth development and have been shown too not only function in mesenchymal cells but also impact epithelial differentiation. These functions are mediated via type I and type II receptors, which facilitate downstream Smad phosphorylation. These Smads, including Smad1/5/8, are essential for normal development of these tissues and for overall survival in some cases. Indeed, elimination of Smad1 expression in a murine knockout (KO) model was shown to be embryonically lethal at E10.5 due to extra-embryonic ectoderm and mesoderm dysplasia^40^. Furthermore, embryonic lethality was also observed in Smad5 KO mice as a result of multiple embryonic and extraembryonic defects^41^. Retardation of hair morphogenesis and failure to differentiate were also observed in Smad1 and 5 double KO mice^42^. A Smad4 conditional KO mouse model (K14-SMAD4) was recently shown to have dental cusp patterning defects, and palatal fusion was downregulated by p38 MAP inhibition^43^.

Furthermore, changes in BMP type I and type II receptor expression and/or function have also been shown to have major downstream effects on morphology. For example, a BMPRIB mutation was previously associated with brachydactyly type A1, an autosomal dominant disorder primarily characterised by hypoplasia/aplasia of the middle phalanges of digits 2–5 44. Missense mutations in the GDF5 binding region of this receptor resulted in reduced phosphorylation of Smad1/5/8 and were also associated with brachydactyly^45^. These symptoms all appear to be induced by inhibition of endochondral ossification in the mesenchyme. In contrast, epithelial BMPRIA regulates the differentiation of progenitor cells of the inner root sheath and hair shaft^46^.

BMP-stimulated non-Smad pathways have recently been the main focus in the literature. In particular, BMP4 has been shown to inhibit the p38 and MAPK pathways via BMPRIA during the self-renewal of embryonic stem cells^47^. In contrast, GDF5 induced phosphorylation of p38 MAP kinase and extracellular signal-regulated kinase (ERK), but not that of c-Jun N-terminal kinase (JNK) in a chondrogenic cell line^48^. Although various BMP and GDFs, along with their receptors, have been linked to the development of a myriad of tissues via both Smad- and non-Smad-mediated pathways, the occurrence of conflicting signalling events has actually made the process more difficult to understand, and the mechanisms underlying the development of certain tissues are still currently unknown.

Tooth development is known to involve a wide range of proteins, including AMEL and AMBN. In fact, AMEL knockout mice were observed to have defects in the cementum and abnormal osteoclast formation mediated by changes in receptor activator of nuclear factor kappa-B ligand (RANKL) expression in the tooth root^49^,^50^. Furthermore, AMEL was shown to inhibit the expression of RANKL and macrophage-colony stimulating factor (M-CSF) in osteoblasts^51^. AMEL and AMBN have also largely been used as ameloblast differentiation markers during tooth development. However, other BMPs and GDFs have also been suggested to play a role, although their full functions and interactions are largely unknown. In the present study, we sought to better understand the function of GDF5 during ameloblast differentiation, particularly in relationship to BMP2. To our knowledge, this is the first study to implicate a function for GDF5 during ameloblast differentiation.

We first sought to determine the expression pattern of GDF5 and its corresponding receptors in the tooth germ. BMPRIA, BMPRIB, and BMPRII were detected in the ameloblasts, whereas GDF5 was observed in the stellate reticulum and the ameloblasts in the cuspis-dentis area. Furthermore, the developmental stages with the highest levels of GDF5 expression were those with the highest levels of BMPRIA, BMPRII, ACVRIIA and ACVRIIB, suggesting that GDF5 functions during ameloblast differentiation via these receptors. To fully elucidate the underlying mechanism, we used the SF2 dental epithelial cell line to first confirm that BMP2 stimulates the production of enamel matrix proteins (e.g., AMEL and AMBN) and ameloblast differentiation. We then supplemented the cells with GDF5 to determine its possible function. The effect of GDF5 was weak and it was masked by the effect of BMP. These data suggest that excess GDF5 does not strongly influence enamel formation in ameloblasts and that the endogenous level of GDF5 may be sufficient for its maximum effect in the dental epithelium. However, excess GDF5 inhibited BMP2-induced AMEL and AMBN expression in SF2 cells, suggesting that affinity of GDF5 to their receptor is lower than that of BMP2.

Several point mutations have been detected in GDF5, including W414R^33^, N445T^34^, N445K^34^, and S475N^32^, and these mutations cause downstream issues in calcification. Here, we further investigated the GDF5^Rgsc451^ mouse, which has brachydactyly and ankylosis in the distal limb skeleton in heterozygotes and ankylosis of the knee joint and osteoarthritis of the elbow joint caused by cartilage dysgenesis^39^. Thus, it seems that this GDF5 mutation disrupts bone morphogenetic signalling. This was confirmed in the present study, in which the calcification of the cranial sphenoid bone and mandibular condyle was mildly suppressed. However, we were particularly interested in the enamel hyperplasia (in contrast to the normal dentin and root formation) that we observed in these mice, particularly because GDF5 has been shown to be involved in root formation, and its influence on periodontal ligament and alveolar bone formation has been previously studied^18^,^52^. The minimal effect of this point mutation on dentin differentiation therefore likely indicates that GDF5 does not function, or at least functions differently, in mesenchymal cells during tooth development compared to dental epithelial cells. This conclusion initially appears to contrast with previous reports indicating a role for GDF5 during root formation. However, we observed that GDF5, as well as BMPRIA and BMPRII, was upregulated in the molar tooth germ of postnatal mice at a stage when enamel calcification was nearing completion and root formation was initiating. Therefore, what may have been perceived as a role in root formation at this time point may actually have represented a role in late-stage enamel calcification.

Amino acid 408 of GDF5 in the GDF5^Rgsc451^ mouse is mutated from W to R. This residue appears to be located at the protein surface in the tertiary structure, potentially indicating a role in molecular binding. The W408R amino acid substitution in the mouse corresponds to the W414R substitution in humans, and the region harbouring this substitution in humans was previously suggested to bind to Noggin and BMPRI^34^. The N445 substitution also appears to result in binding to Noggin and BMPRI, while also abolishing the BMP inhibitor function of Noggin, and has been associated with multiple synostosis syndrome^34^. W408R in the mouse may thus have a function similar to that of N445. These molecules associated with BMP signalling, including BMP2, BMP4, BMPRI, BMPRII, Noggin, and p-Smad1/5/8, were all previously shown to be strongly expressed in the dental epithelium^53^ and appear to play essential roles^54^. For example, Noggin inhibits tooth germ differentiation, and enamel is absent in the K14-Noggin transgenic mouse^55^,^56^. In contrast, the BMPRIA conditional KO mouse has defective tooth development and presents interruption of epithelial BMP signalling via BMPRIA depletion, which switches the differentiation of crown epithelia into the root lineage^26^. It has also been suggested that follistatin, a known BMP inhibitor, may also be involved in crown and root border formation^57^. In the present study, we chose to focus on the downstream phosphorylation of Smad1/5/8. Mutant, but not wild-type, GDF5 enhanced the phosphorylation of Smad1/5/8 by BMP2. It is possible that the W408R GDF5 mutant lost the antagonising function of GDF5 on BMPRI, resulting in enhanced BMP2 signalling, because excess GDF5 inhibited BMP2-induced ameloblast differentiation (Fig. 4). Another possibility is that mutant GDF5 binds to Noggin, similar to the human N445T mutant, preventing this repressor from inhibiting BMP2 signalling. Furthermore, the increase in BMP signalling resulted in an increase in intracellular Smad1/5/8 phosphorylation (and possibly subsequent activation of Runx2) in the dental epithelium, inducing excessive enamel calcification. A recent study based on structural analysis of GDF5 and BMPRIA showed inhibition of BMP2-induced alkaline phosphatase expression by increasing concentrations of GDF5 protein in the chondrogenic cell line ATDC5. Half-maximal inhibitory concentrations (IC50) of 70 nM and 5 nM were observed for wild-type GDF5 and GDF5 R57A, respectively^31^. These conclusions are supported by previous studies investigating these relationships^58^,^59^. However, the effect of Noggin effect on mutant GDF5 including its affinity to BMPRI should be confirmed in future experiments.

In conclusion, in the present study we analysed the expression of wild-type GDF5 and various BMPRs to identify the role of GDF5 during tooth development. In doing so, we identified a novel role for the W408R GDF5 mutation in accelerated enamel formation in mice. Although this study not only advances the understanding of basic ameloblast differentiation during tooth development but also provides a possible therapeutic use for enamel regeneration.

## Methods

### Mouse strain

Paraformaldehyde-fixed bodies from 8-week-old male GDF5^Rgsc451^ mice were generously provided by the RIKEN Bioresource Center through the National BioResource Project of the MEXT, Japan^39^. Using these mice, we subsequently identified a mutant line, termed M100451, which exhibited a brachypodism phenotype (posted on the website: http://www.gsc.riken.jp/mouse/). Similarly fixed samples from 8-week-old male C57BL/6J mice were used as the controls in all *in vivo* experiments. Tooth germ cells were also isolated from C57BL/6J mice at various stages of development (E13 to postnatal day 7). All experiments were obeyed the Law Concerning the Conservation and Sustainable Use of Biological Diversity through Regulations on the Use of Living Modified Organisms, and genetic recombination experiments were approved by the Tohoku University Center for Gene Research. All experiments were performed in accordance with the approved guidelines.

### RNA isolation and real-time PCR

Total RNA was prepared from non-transfected and transfected SF2 cells as well as tooth germ cells isolated from C57BL/6J mice using TRIzol^®^ (Life Technologies, NY, USA). First-strand cDNA was synthesised at 50 ˚C for 50 minutes using oligo(dT) or random primers with the Super Script III First-Strand Synthesis System (Life Technologies). PCR was performed with SYBR Select Master Mix (Applied Biosystems, CA, USA) and a Step One Plus Real-Time PCR system (Applied Biosystems). The following forward (F) and reverse (R) primers were utilised for RT-PCR: rat BMPRIA (NM_030849.1; F: 5′-TTGCGGCCAATCGTCTCTAA-3′, R: 5′-AAGGCATCAAAAGCCCACAT-3′), rat BMPRIB (NM_001024259.1; F: 5′-ACGGCCTTCATTCCCCAATC-3′, R: 5′-TCCGTGTTTCTGGGTTCCTC-3′), rat BMPRII (NM_080407.1; F: 5′-CCACGGACATGCCTTCAGTT-3′, R: 5′-GATTCTGGGAAGCAGCCGTAG-3′), rat ACVRIIA (NM_031571.2; F: 5′-GTTACACCGAAGCCACCCTA-3′, R: 5′-ACAGGAGGGTAGGCCATCTT-3′), rat ACVRIIB (NM_031554; F: 5′-GGAGTGACCCAGTCACCAGT-3′, R: 5′-GATGCTTCTGACAACCAGCA-3′) and rat noggin (NM_012990.1; F: 5′-TGTGGTCACAGACCTTCTGC-3′, R: 5′-GTGAGGTGCACAGACTTGG-3′). Primers for AMEL (NM_001271073.1; F: 5′-GTCACACCCTTCAGCCTCATC-3′, R: 5′-GTGTTGGGTTGGAGTCATGGA-3′) and AMBN (NM_012900.1; F: 5′-TGTAGGTCCCTTCTTGCTTCC-3′, R: 5′-TGCCTAAGACAGCTACATGCT-3′) were used for real-time PCR. Expression of each gene was normalised to GAPDH expression. In experiments comparing experimental conditions to a control condition (or the earliest time point), expression in the control sample was set to 1.0 and expression in the experimental samples were determined as the fold change relative to the control using the ΔΔCT method (n = 5). Statistical analysis of gene expression was performed using Student’s *t*-test, with p < 0.05 considered significant. The PCR products were collected from the samples after 27–32 cycles and then electrophoresed on 2.5% agarose gels. The gels were stained by SYBR Safe DNA gel stain (Life Technologies) and bands were detected using a UV-transilluminator.

### Immunohistochemistry

Paraffin-embedded P1 lower jaws (n = 5) were sliced to a thickness of 8 μm. Selected sections were then soaked in L.A.B. Solution (Polysciences, PA, USA) for 5 min for antigen activation, followed by incubation in 5% BSA/PBS for 1 h. After incubation with the primary antibodies for BMPRIA (sc-20736; Santa Cruz Biotechnology, CA, USA), BMPRIB (sc-25455; Santa Cruz Biotechnology), or BMPRII (MABD171; Merck Millipore, MA, USA), expression was detected using Alexa488- or Alexa594-conjugated secondary antibodies (Life Technologies). A fluorescence microscope (BZ-8000, KEYENCE Co, Osaka, Japan) was used for image analysis. Images were prepared using a BZ analyzer (KEYENCE) and Photoshop (Adobe Systems, CA, USA).

### GDF5 expression plasmid constructs

The rat GDF5 (XM_001066344.2) gene was ligated into the pCMV/hygro-His expression plasmid (RG80131-G-H; Sino Biological Inc., Beijing, China). Amino acid 408 in the GDF5 gene was then mutated from tryptophan to arginine using a QuickChange Lightning Mutagenesis Kit (Agilent Technologies, Santa Clara, CA, USA). The modified mutant GDF5 (mut-GDF5) was confirmed by sequencing.

### Cell culture and transfection

SF2 cells, a rat dental epithelial cell line, were established as previously described.^38^ Cells were cultured in DMEM/F12 medium (Life Technologies) containing 10% foetal bovine serum (Life Technologies) and an antibiotic cocktail (Gibco, NY, USA) at 37 °C in an atmosphere containing 5% CO_2_. SF2 cells were cultured with 100 ng/ml recombinant human BMP2 (Wako, Japan) or 100, 1000 ng/ml mouse GDF5 (Pepro Tech, NJ, USA) and were collected for RT-PCR analysis after a 24 h incubation period.

For plasmid transfection, SF2 cells were cultured to density of 0.1 × 10^5^ cells per well in a 6-well plate without BMP2. Prior to transfection with the wild-type GDF5 (wt-GDF5) or modified mut-GDF5 expression plasmid, the medium was replaced with OPTI-MEM (Life Technologies). The cells were transiently transfected using ViaFect (Promega, WI, USA) according to the manufacturer’s protocol. Following transfection, fresh DMEM/F12 culture medium containing 10% foetal bovine serum and antibiotics was added, and the cells were incubated for an additional 24 h with 100 ng/ml BMP2.

### Structural analysis of mouse GDF5

The amino acid sequences of mouse BMP2-15, including GDF5/BMP14, were examined using the NCBI database (http://www.ncbi.nlm.nih.gov/protein/). Identical amino acids were coloured yellow and amino acids with high similarity were coloured green after the amino acids were aligned. Based on this alignment, the tertiary structure was subsequently analysed with UCSF CHIMERA (http://www.cgl.ucsf.edu/chimera/). Amino acid 408 was identified in the tertiary structure of mouse GDF5, and a three-dimensional reconstructed image was generated using software. The region of GDF5 that bound to BMPRIB was traced and coloured (light blue) using Illustrator (Adobe Systems, San Jose, CA).

### Micro-computed tomography (micro-CT) scanner imaging

Skulls of 8-week-old wild-type and GDF5^Rgsc451^ mice were analysed by micro-CT at the Kureha Special Laboratory (Iwaki, Japan). The mandibles were imaged, and the mineralisation volumes (mm^3^) of enamel and dentin were calculated from the tomographically sliced sections. The first molar root width and length were also measured. The mineral volumes and root distances of WT were defined as 1.0, and the relative amounts for the mutant mice were calculated accordingly.

### Western blotting

SF2 cells transfected with wt-GDF5 or mut-GDF5 were plated in 6-well plates at a concentration of 1 × 10^5^ cells per well and incubated for 48 h. The cells were then cultured without serum-containing medium for 2 h, followed by treatment with 100 ng/ml BMP2 for 0, 5, 15, 30, or 60 min at 37 °C. Thereafter, the cells were washed twice with ice-cold 1 mM sodium orthovanadate (Sigma) in PBS, lysed with Nonidet P-40 buffer supplemented with a proteinase inhibitor mixture (Sigma), and centrifuged. The supernatants were then transferred to a fresh tube. The cell lysates were separated with 4–12% gradient SDS-PAGE and analysed by western blotting. The blotted PVDF membrane was incubated with Smad5 or phospho Smad1/5/8 primary antibodies (Cell Signaling Technology, Beverly, MA, USA). The signals were detected with an ECL kit (GE Healthcare, Milwaukee, WI, USA) after treatment with a rabbit HRP-conjugated secondary antibody. Images were visualised using the ImageQuant LAS 4000 Mini image analysis system (GE Healthcare, Life Science).

## Additional Information

**How to cite this article**: Liu, J. *et al.* Mutant GDF5 enhances ameloblast differentiation via accelerated BMP2-induced Smad1/5/8 phosphorylation. *Sci. Rep.* **6**, 23670; doi: 10.1038/srep23670 (2016).

## Supplementary Material

Supplementary Information
