# Supplementary material for: Mutant GDF5 enhances ameloblast differentiation via accelerated BMP2-induced Smad1/5/8 phosphorylation
Source: Sci Rep. 2016 Mar 31;6:23670. doi: 10.1038/srep23670 (PMC4814822; doi:10.1038/srep23670)
Supplement: Supplementary Information [file srep23670-s1.pdf]

Mutant GDF5 enhances ameloblast differentiation via accelerated BMP2-induced Smad1/5/8 phosphorylation

Jia Liu[1 2 3‡], Kan Saito[2‡*], Yuriko Maruya[2,4‡], Takashi Nakamura[2], Aya Yamada[2], Emiko Fukumoto[2], Momoko Ishikawa[2], Tsutomu Iwamoto[2], Kanako Miyazaki[5], Keigo Yoshizaki[5], Lihong Ge[1]*, Satoshi Fukumoto[2]

Supplemental figure

a

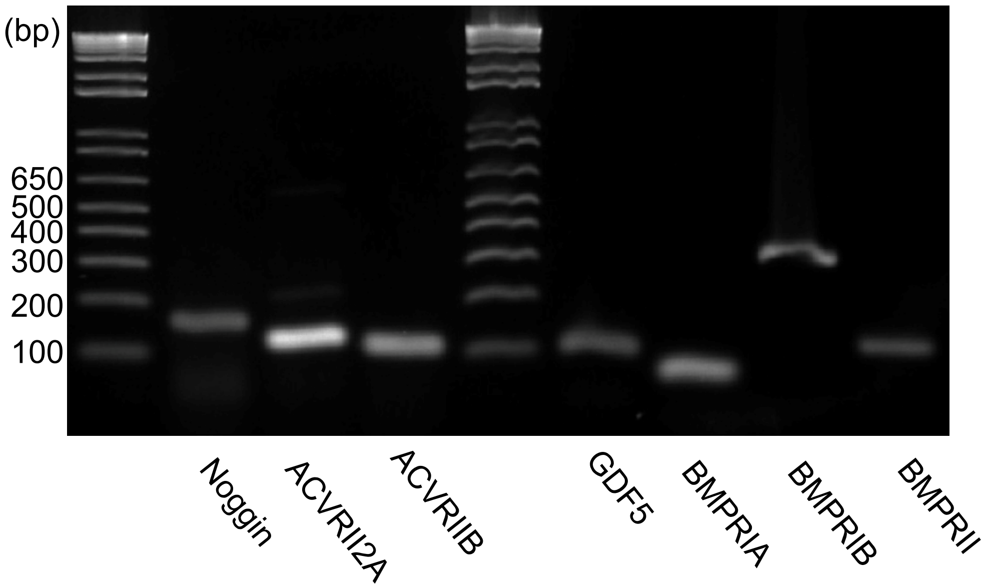

Supplemental figure

b

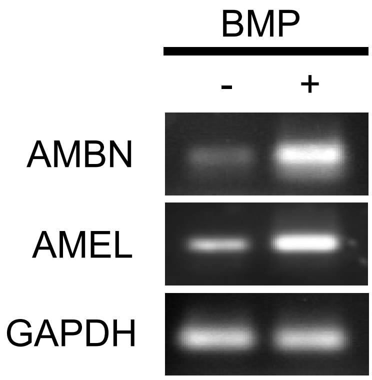

Supplemental figure

c

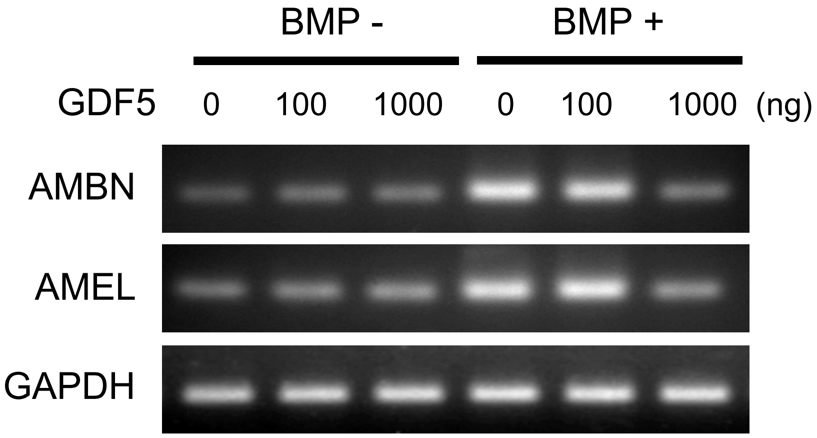

Supplemental figure. Electrophoretic pattern of RT-PCR. The results for GDF5, BMPRs, ACVRs and noggin expressions in mouse tooth germ cells from E13 to P7 were confirmed by electrophoresis (a). SF2 cells were cultured with 100ng/ml BMP2 for 24 h, and the expression of AMEL and AMBN was analysed by RT-PCR (b). SF2 cells were cultured with 0, 100 ng/ml BMP2 and 0-1000 ng/ml GDF5 for 24 h. AMEL and AMBN expression was detected by RT-PCR (c).
